# Implant-Supported Prosthesis Is a Viable Treatment Alternative for American Society of Anesthesiology Physical Status 3 Individuals—A Retrospective Cohort Study

**DOI:** 10.3390/jcm11072002

**Published:** 2022-04-02

**Authors:** Daya Masri, Hiba Masri-Iraqi, Sarit Naishlos, Evgeny Weinberg, Vadim Reiser, Liat Chaushu

**Affiliations:** 1 Department of Oral and Maxillofacial Surgery, Rabin Medical Center, Petach-Tikva 4941492, Israel; vadik.raiser@gmail.com; 2 Department of Endocrinology, Rabin Medical Center, Petach-Tikva 4941492, Israel; iraqihiba@yahoo.com; 3 Department of Pedodontics, The Maurice and Gabriela Goldschleger School of Dental Medicine, Tel Aviv University, Tel Aviv 6997801, Israel; river554@gmail.com; 4 Department of Periodontology and Oral Implantology, The Maurice and Gabriela Goldschleger School of Dental Medicine, Tel Aviv University, Tel Aviv 6997801, Israel; evgenywein@gmail.com

**Keywords:** American Society of Anesthesiologists Physical Status (ASA PS), early implant failure (EIF), augmented bone, implant brand

## Abstract

Background: Within medicine, it is common to use risk prediction tools towards clinical decision making. One of the most widely accepted assessment tools is the American Society of Anesthesiologists Physical Status (ASA PS) classification. Oral and maxillofacial procedures performed in an ambulatory setting would be considered low risk for the procedure itself. However, little is known concerning the impact of ASA PS on surgical outcomes. The aim of the present research was to evaluate the effect of ASA PS classification on early implant failure (EIF). Methods: Retrospective cohort study based on dental records. All treatments were performed by experienced oral and maxillofacial surgeons and experienced prosthodontists. Inclusion criteria: ASA physical status 1,2,3, consecutive individuals. Variables included the following: age, gender, implant location, implant length, implant width, smoking, and early implant failure. Results: Univariate tests at the patient level showed no statistically difference between the different classifications of ASA PS (1,2,3). Multivariate model using logistic regression at individual level showed that two factors were found to be associated with an increased risk for EIF—augmented bone and implant brand. Conclusions: ASA PS 3 is not a contraindication for implant-supported prostheses. EIF in ASA PS 3 is not significantly different from ASA PS 1,2. In contrast, factors such as bone augmentation and implant brand might be significant risk factors for EIF, regardless of ASA PS.

## 1. Introduction

Medical improvement and the extension of life expectancy constantly increase the percentage of the elderly population. Larger numbers of individuals, with more complicated systemic comorbidity and wide medication intake will require dental care [1,2]. Moreover, predicting the impact of the physical status on surgical outcome is becoming more important [3].

Implant dentistry has a crucial part in modern dentistry. More individuals require fixed restorations, making implant use popular. Thus, understanding the impact of the physical status on the ultimate fate of installing dental implants is compulsory.

Within medicine, it is common to use risk prediction tools towards clinical decision making [3]. One of the most widely accepted assessment tools is the American Society of Anesthesiologists Physical Status (ASA PS) classification. Oral and maxillofacial procedures performed in an ambulatory setting would be considered low risk for the procedure itself; however, little is known concerning the impact of ASA PS on surgical outcomes [4,5].

Implant failures can be divided into early and late according to failure time. Late implant failures (>12 months after loading) are associated with moderate to severe bone loss; a larger number of failed implants per patient; higher prevalence in males; and occurring mostly in posterior areas [6]. Implants may alter the levels of inflammation of peri-implant tissues. Implant survival may be altered by peri-implant molecules and proteins in sulcus fluids. Recent evidence demonstrates that peri-implant marginal bone loss progression is statistically correlated to sulcus fluid volume, IL-6, Il-1b concentrations, and peri-implant sulcular fluid levels of metalloproteinase-8 [7,8].

Early implant failures (EIF) (<12 months after loading) are associated with minimal bone loss; support single crowns; and higher prevalence in females.

A 10-year retrospective study evaluated the long-term reliability, survival rate, and mechanical and biological complications of single-crown implant rehabilitations with two different types of fixture-abutment connections: screw-retained abutments (SRAs) with internal hexagonal connection and cemented retained abutments (CRAs). Although complications occurred, the results from this 10-year retrospective study showed that these two methods have positive long-term follow-up. Marginal bone loss was significantly greater for the SRA vs. CRA group [9].

Biological and technical failures of implants may occur. The abutment screw fracture or loosening represents a rare but quite unpleasant failure. An analysis and structural examination of screw thread or abutment demonstrated many alterations and deformations in the concavities and convexities of screw threads [10].

EIF can be influenced by variety of factors—smoking, implant location, gender, implant length, implant brand, number of implants, immediate installation after extraction, need of bone augmentation, non-submerged healing, periodontal disease, the clinician, medication intake, and ASA PS [11,12].

The aim of the present research was to assess EIF in cohorts with different ASA PS classification. The null hypothesis was that EIF in ASA PS class 1,2 is lower compared to EIF ASA PS 3.

## 2. Material and Methods

The present retrospective, cohort study is based on dental records of the Department of Oral and Maxillofacial Surgery, Rabin Medical Center, Campus Beillinson, Israel. All treatments were performed by experienced oral and maxillofacial surgeons and prosthodontists.

The study protocol was approved by the ethics committee of the Rabin Medical Center, Campus Beilinson, Israel (0674-19rmc). The present script complies with the STROBE guidelines [13]. Dental records of all individuals included were extracted and manually screened twice by 2 examiners (DM and LC).

### 2.1. Patient Population

Inclusion criteria: all consecutive patients who had received an implant between January 2013 and December 2018; available documentation; and a minimum follow-up of 12 months following prosthetic delivery.

Exclusion criteria: history of head and neck cancer and/or history of receiving radiation therapy to the facial area and/or immune compromised patients due to immunosuppressant medication; heavy smokers; and untreated periodontal disease.

### 2.2. Data Collection

(1) Age
(2) Gender
(3) Physical status according to American Society for Anesthesiology (ASA) [3]
(4) Implant dimensions (length, diameter (mm))
(5) Bone grafting before or at the same time as implant placement (yes/no)
(6) Number of implants placed
(7) EIF—lack of osseointegration up to 12 months after prosthesis delivery and occlusal loading (yes/no; primary outcome variable).

Three types of implants were installed: MPI (Ditron Dental, Ashkelon, Israel) sand-blasted and acid-etched surfacing, SCREW-VENT (Warsaw, IN, USA), hydroxyapatite-blasted surfacing, LANCE PLUS (MIS, Israel), sand blasted, and acid-etched surfacing.

### 2.3. Statistical Analysis

The data were analyzed using SPSS software version 25 (IBM Corporation, Armonk, NY, USA). Descriptive statistics were performed using means and standard deviations for the continuous variables and frequencies for the discrete variables. Univariate correlations were performed using the chi-square ($\chi^{2}$) test. Tests between independent samples were performed using the Mann–Whitney U test. Significance was considered for a *p*-value lower than 5%. Continuous variables did not distribute normally; therefore, non-parametric statistical procedures were used when analysing these data.

## 3. Results

### 3.1. Demographics at Patient Level

The cohort (*n* = 792) comprised 37.2% males and 62.8% females (Table 1A). The participants comprised the following: 58% were 65 years old or lower, while 33.4% were 66–79.9 years old. The remaining 8.6% were 80 years old or older. Five-point one percent of the cohort were smokers. Ditron was given to 13.6% of the cohort, MIS implants were given to 25.8%, and Zimmer implants were given to 60.6%. In 40.8%, patient implants were placed in pristine and in 59.2% patients with augmented bones. Physical status distribution was 32.6%, 34.8%, and 32.6% for ASA 1–3, respectively. At least one EIF per patient was noted in 14.4% of the individuals.

### 3.2. Demographics at Implant Level

All data were likewise measured at the implant level (*n* = 2971) (Table 1B). With regard to implant distribution, 57.7% of the implants were placed in patients at the age of 65 or lower, 34% were in patients between 66 and 79.9 years, and the remaining 8.3% were provided to those at the age of 80 or older. Smokers received 6.6% of the implants. Implant distribution was 13.6%, 26.4%, and 60% for Ditron, MIS, and Zimmer. Augmentation was performed for 61.2% implants, while 38.8% were placed in pristine bone. Physical status distribution was 27.3%, 39.7%, and 33% for ASA 1–3, respectively. Implant location was 15.3% anterior maxilla, 18.4% premolar maxilla, 15% posterior maxilla, 13.6% anterior mandible, 17% premolar mandible, and 20.4% posterior mandible. EIF was recorded for 3.8% of the implants. For a complete description, see Table 1A for patient level descriptive statistics and Table 1B for implant level descriptive statistics.

### 3.3. Univariate Analysis

To test each independent variable with the failure or success of the implant, univariate tests were conducted at the patient level (Table 2A) and at the implant level (Table 2B).

As can be observed at the patient level (Table 2A) a significant relation between the type of implant and failure ($\chi^{2}\left(2\right)=9.57,p=0.01$) was found. Ditron implants’ success rates (12.5%) were lower than their failure rates (20.2%). Similarly, Zimmer implants’ success rates (60%) were lower than their failure rates (64%). Mis-implants, on the other hand, had a higher success rate (27.4%) than their failure rate (15.8%).

As observed from the implant level at Table 2B, a significant relation was found between implant type and failure ($\chi^{2}\left(2\right)=9.15,p=0.01$). Ditron implants’ failure proportion (20.2%) was higher than their success proportion (13.3%). Similarly, Zimmer implants failure (64%) was higher than success (59.9%). However, more MIS implants succeeded (26.8%) than failed (15.8%). A significant relation was found between augmentation and failure ($\chi^{2}\left(1\right)=14.22,p<0.001$). Implants placed in pristine bone success (39.5%) was higher than failure (21.9%); on the other hand, implants placed in augmented bone success (60.5%) was lower than failure (78.1%). A significant relation was found between ASA and failure ($\chi^{2}\left(2\right)=6.33,p=0.04$). Implants with ASA 1 had a failure (36.8%) higher than their success (27%). However, implants with ASA 2 had a failure (30.7%) lower than their success (40%). Similarly, implants with ASA 3 had a failure (32.5%), which was lower than their success (33%). A significant relation was found between anterior maxilla and failure ($\chi^{2}\left(1\right)=9.20,p=0.002$). Anterior maxillary implants success (15.7%) was higher than their failure (5.3%). A significant relation was found between premolar maxilla and failure ($\chi^{2}\left(1\right)=7.37,p=0.01$). Premolar maxillary implants’ success (18.8%) was higher than their failure (8.8%). A significant relation was found between posterior maxilla and failure ($\chi^{2}\left(1\right)=48.14,p<0.001$). Posterior maxillary implants success (14.1%) was lower than failure rate (37.7%). A significant relation was found between anterior mandible and failure ($\chi^{2}\left(1\right)=14.04,p<0.001$). Anterior mandibular implants success (13.2%) was lower than failure (25.4%). A significant relation was found between premolar mandible and failure ($\chi^{2}\left(1\right)=4.54,p=0.03$). Premolar mandibular implants success (17.3%) was higher than failure (9.6%). Lastly, a significant relation was found between posterior mandible and failure ($\chi^{2}\left(1\right)=4.82,p=0.03$). Posterior mandible implants’ success rate (20.7%) was higher than failure (12.3%). Implants in the anterior mandible and implants in the posterior maxilla failed more than other areas in the jaws.

### 3.4. Multivariate Analysis

A Logistic regression model at the implant level showed that the independent variables significantly predicted failure (χ^2^(16) = 99.69, *p* < 0.001), while explaining about 12% of total variance in failure. The model is well-fit to the data (χ^2^(8) = 4.38, *p* = 0.82) while classifying about 96.1% of the total observations.

It was found that implants in ASA PS 2 predicted a higher probability for failure in comparison with ASA1 (OR = 0.54, *p* = 0.01). Moreover, the odds of implant failure when the type of implant was Ditron are 2.30 times as likely than the odds for implant failure for MIS implant types (OR = 2.3, *p* = 0.002). Lastly, the odds of failure for implant failure on augmented bone was 2.14 times higher than implants placed in pristine bone (OR = 2.14, *p* = 0.002). For complete regression coefficients, see Table 3A.

A logistic regression model at the patient level showed that the independent variables significantly predicted failure (χ^2^(10) = 32.62, *p* < 0.001), while explaining about 7.4% of the total variance in failure. The model is well-fit to the data (χ^2^(8) = 2.53, *p* = 0.96) while classifying about 85.6% of the total observations.

It was found that the odds of implant failure when the type of implant is Ditron are 2.66 times as likely than the odds for implant failure for MIS implant types (OR = 2.66, *p* = 0.01). Similarly, the odds of implant failure when the type of implant is Zimmer are 1.80 times as likely than the odds for implant failure for the MIS implant brand (OR = 1.80, *p* = 0.04). For complete regression coefficients, see Table 3B.

## 4. Discussion

Our medical center is a referral for patients with challenging systemic comorbidities and patients with extremely complex dental cases. Consequently, we were able to assess the impact of systemic factors and drug intake on EIF. The data collected in the present study were used to assess the impact of different cofounders on EIF. However, the interest was in the effect of physical status. ASA PS classification was used to compare clinical outcomes between individuals.

To the best of our knowledge, only a few studies investigated the impact of ASA PS on dental implants failure in general and EIF in particular. Casino et al., 1988 were the first individuals taking ASA PS variable into account. ASA PS 3 (severe systemic disease) individuals had statistically significant higher failure rates than ASA PS 1,2 (healthy and mild systemic disease) (*p* < 0.0011) [14].

Zinser et al., 2012 assessed the predictors of implant failure after maxillary sinus augmentation. They reported that advanced classes (ASA PS 2,3) are a significant predictor (*p* < 0.002) for failure. Compared to healthy patients (ASA PS 1), the increased risk of implant failure was 2.7 for ASA PS 3 and 1.97 for ASA PS 2 patients times [15].

The same trend was observed by Conrad et al., 2011 who showed statistically significant increase (*p* < 0.001) in implant failure in ASA PS 3 individuals with an increased risk of implant failure of 8.83 times compared to ASA PS 1 [16]. Drawbacks of those studies include the assessment of ASA PS impact only on maxillary posterior area with and without sinus augmentation without addressing the failure risk in the other jaw areas. Another limitation for these two studies is that, in the study of Conrad et al., 2011, a small sample of implants was reported, while in the study of Zinser et al., 2012, patients’ sample sizes were not included.

In the present study, two factors were found to be associated with an increased risk for EIF—augmented bone and implant brand. There have only been a few published studies that compared implant survival between augmented versus pristine bone. The results have been contradictory and are often affected by various limitation. A systematic review [17] and three retrospective studies [18,19,20] found no difference between the survival of implants in pristine versus augmented bone, which challenges the results of the present study. Other studies found differences in survival rates between implants placed in grafted vs. pristine bone [19,20,21]. Sesma et al. [21] found a significant association between implant failure and the presence of bone graft in the implant area. The confidence intervals associated with the failure rates were large, indicating the low precision of the results. Two other studies found higher success rates for implants placed in the grafted maxillary sinuses vs. non-grafted posterior maxilla [19,21]. Olson et al. attributed the results to the use of high-diameter implants in the grafted sites [22]. Shortcomings of these studies include insufficient follow up, small sample size to allow statistical analysis, and a lack of control of the confounder.

The results of the present study are based on a relatively large patient (*n* = 792) and implant sample (*n* = 2971), and the implants are distributed in the different areas of the jaws (anterior mandible, premolar mandible, posterior mandible, anterior maxilla, premolar maxilla, and posterior maxilla). In the present study, no statistically significant increase in EIF was associated with high classes of ASA PS at both patient and implant levels in univariate and multivariate analysis, contrary to the literature published so far. Further prospective studies in this field are required to elucidate which comorbidities and drugs may influence EIF, both positively and negatively.

Implant brand has long been under the dispute of many studies. In this specific cohort, the implant brand was associated with an increased risk for EIF.

The limitations of the current study include the following: retrospective, only one treatment center, and limited implant brands and augmentation material. The strengths of the present study include the following: a significant number of individuals, only specialist-provided treatment, and standard working protocol. Further studies are needed to validate the effect of ASA PS on EIF, late failures, immediate implant placement, and different loading protocols.

## 5. Conclusions

It can be concluded that within the limitations of the present study, ASA PS 3 is not a contraindication for implant-supported prostheses. Moreover, the EIF in all three ASA PS 1-3 is not significantly different. Future studies should assess the effect on different osseointegration and loading protocols. In contrast, factors such as bone augmentation and implant brand might be significant risk factors for EIF, regardless of ASA PS.

## Figures and Tables

**Table 1:** (**A**): Demographics and baseline clinical characteristics of the cohort at patient level (*n* = 792). (**B**): Demographics and baseline clinical characteristics of the cohort at implant level (*n* = 2971).

(**A**)				
	**M**	**SD**	***n***	**%**
Demographic Characteristics				
Gender				
Male			295	37.2
Female			497	62.8
Age Group				
65 or less			459	58
66–79.9			265	33.4
80 or more			68	8.6
Smoke			40	5.1
Clinical Characteristics				
Implant Brand				
Ditron			108	13.6
MIS			204	25.8
Zimmer			480	60.6
Augmentation				
Pristine			323	40.8
Augmented			469	59.2
ASA				
ASA 1			258	32.6
ASA 2			276	34.8
ASA 3			258	32.6
Failure			114	14.4
(**B**)				
	**M**	**SD**	***n***	**%**
Demographic Characteristics				
Age Group (years)				
65 or less			1715	57.7
66–79.9			1009	34
80 or more			247	8.3
Smoking			195	6.6
Clinical Characteristics				
Augmentation				
Pristine			1153	38.8
Augmented			1818	61.2
ASA				
ASA 1			812	27.3
ASA 2			1178	39.7
ASA 3			981	33
Anterior maxilla			455	15.3
Premolar maxilla			551	18.5
Posterior maxilla			445	15
Anterior mandible			406	13.7
Premolar mandible			506	17
Posterior mandible			608	20.5
Length	11.38	1.60		
Width	3.84	0.31		
Failure			114	3.8

**Table 2:** (**A**). Univariate tests at the patient level. (**B**). Univariate tests at the implant level.

(**A**)						
		**Success**		**Failure**		
**Variable**	**Group**	***n* (%**)	**M ± SD**	***n* (%**)	**M ± SD**	***p*-Value**
Gender	Male	256 (37.8%)		38 (33.3%)		0.36
	Female	422 (62.2%)		76 (66.7%)		
Age Groups	65 or less	391 (57.7%)		68 (59.6%)		0.10
	66–79.9	223 (32.9%)		42 (36.8%)		
	80 or more	64 (9.4%)		4 (3.5%)		
Smoke	No	643 (94.8%)		109 (95.6%)		0.73
	Yes	35 (5.2%)		5 (4.4%)		
Implant Brand	Ditron	85 (12.5%)		23 (20.2%)		0.01
	Mis	186 (27.4%)		18 (15.8%)		
	Zimmer	407 (60%)		73 (64%)		
Bone type	Pristine	298 (44%)		25 (21.9%)		<0.001
	Augmented	380 (56%)		89 (78.1%)		
Augmetation	No	298 (44%)		25 (21.9%)		<0.001
	Yes	380 (56%)		89 (78.1%)		
ASA	ASA 1	216 (31.9%)		42 (36.8%)		0.50
	ASA 2	241 (35.5%)		35 (30.7%)		
	ASA 3	221 (32.6%)		37 (32.5%)		
(**B**)						
		**Success**		**Failure**		
**Variable**	**Group**	***n*** **(%)**	**M ± SD**	***n*** **(%)**	**M ± SD**	***p*-Value**
Age Groups	65 or less	1647 (57.6%)		68 (59.6%)		0.16
	66–79.9	967 (33.8%)		42 (36.8%)		
	80 or more	243 (8.5%)		4 (3.5%)		
Smoke	No	2667 (93.3%)		109 (95.6%)		0.34
	Yes	190 (6.7%)		5 (4.4%)		
Implant Brand	Ditron	380 (13.3%)		23 (20.2%)		0.01
	MIS	765 (26.8%)		18 (15.8%)		
	Zimmer	1712 (59.9%)		73 (64%)		
Bone type	Pristine	1128 (39.5%)		25 (21.9%)		<0.001
	Augmented	1729 (60.5%)		89 (78.1%)		
ASA	ASA 1	770 (27%)		42 (36.8%)		0.042
	ASA 2	1143 (40%)		35 (30.7%)		
	ASA 3	944 (33%)		37 (32.5%)		
Anterior maxilla	No	2408 (84.3%)		108 (94.7%)		0.002
	Yes	448 (15.7%)		6 (5.3%)		
Premolar maxilla	No	2319 (81.2%)		104 (91.2%)		0.01
	Yes	538 (18.8%)		10 (8.8%)		
Posterior maxilla	No	2455 (85.9%)		71 (62.3%)		<0.001
	Yes	402 (14.1%)		43 (37.7%)		
Anterior mandible	No	2481 (86.8%)		85 (74.6%)		<0.001
	Yes	376 (13.2%)		29 (25.4%)		
Premolar mandible	No	2363 (82.7%)		103 (90.4%)		0.03
	Yes	494 (17.3%)		11 (9.6%)		
Posterior mandible	No	2264 (79.3%)		100 (87.7%)		0.03
	Yes	592 (20.7%)		14 (12.3%)		
Implant Length (mm)			11.37 ± 1.60		11.57 ±1.52	0.41
Implant Width (mm)			3.87 ± 0.31		3.84 ± 0.34	0.65

**Table 3:** (**A**). Binary Logistic regression coefficients (at the implant level) to predict implant failure. (**B**). Binary logistic regression coefficients (at the patient level) to predict implant failure.

(**A**)				
	**OR**	**95% CI Lower**	**95% CI Upper**	***p***
**ASA (ASA 2)**	0.54	0.34	0.88	0.01
**ASA (ASA 3)**	0.74	0.44	1.22	0.23
**Age group** **(66–79.9)**	1.05	0.68	1.63	0.83
**Age group** **(80 or more)**	0.53	0.18	1.54	0.24
**Smoke**	0.66	0.26	1.69	0.39
**Implant brand (Ditron)**	2.30	1.20	4.40	0.01
**Implant type (Zimmer)**	1.58	0.92	2.70	0.09
**Bone type (Augmented)**	2.14	1.33	3.44	0.002
**Anterior maxilla**	0.21	0.03	1.49	0.12
**Premolar maxilla**	0.30	0.05	1.99	0.21
**Posterior maxilla**	1.77	0.29	10.94	0.54
**Anterior mandible**	1.30	0.21	8.15	0.78
**Premolar mandible**	0.35	0.05	2.34	0.28
**Posterior mandible**	0.39	0.06	2.51	0.32
**Implant Length(mm)**	1.00	0.87	1.15	0.98
**Implant Width(mm)**	0.78	0.41	1.49	0.45
Note: The reference group for the age group variable is “ages 65 or lower”. The reference group for implant type is “MIS” and the reference group for ASA is “ASA 1”. The reference group for the Augmentation variable is “Pristine”.				
(**B**)				
	**OR**	**95% CI Lower**	**95% CI Upper**	***p***
**Sex (Female)**	1.21	0.78	1.88	0.39
**Age group** **(66–79.9)**	1.04	0.65	1.67	0.87
**Age group** **(80 or more)**	0.53	0.18	1.58	0.26
**Smoke**	0.90	0.34	2.42	0.83
**Implant brand (Ditron)**	2.66	1.32	5.36	0.01
**Implant brand (Zimmer)**	1.80	1.03	3.15	0.04
**Augmentation (Yes)**	2.68	1.65	4.36	<0.001
**ASA (ASA 2)**	0.76	0.45	1.29	0.32
**ASA (ASA 3)**	0.97	0.56	1.67	0.90
Note: The reference group for the age group variable is “ages 65 or lower”. The reference group for implant type is “MIS”, and the reference group for ASA is “ASA 1”.				

## Data Availability

The data presented in this study are available on request from the corresponding author. The data are not publicly available due to ethics.

## References

[1] Liotta G., Canhao H., Cenko F., Cutini R., Vellone E., Illario M., Kardas P., Poscia A., Sousa R.D., Palombi L. (2018). Active ageing in Europe: Adding healthy life to years. Front. Med..

[2] Central Bureau of Statics, Statistical Abstract of Israel 2019. https://www.cbs.gov.il/en/publications/Pages/2019/Statistical-Abstract-of-Israel-2019-No-70.aspx.

[3] Irlbeck T., Zwißler B., Bauer A. (2017). ASA-Klassifikation: Wandel im laufe der zeit und darstellung in der literatur. Anaesthesist.

[4] de Jong K.J.M., Oosting J., Abraham-lnpijn L. (1993). Medical risk classification of dental patients in The Netherlands. J. Public Health Dent..

[5] Maloney W.J., Weinberg M.A. (2008). Implementation of the American Society of Anesthesiologists Physical Status classification system in periodontal practice. J. Periodontol..

[6] Manor Y., Oubaid S., Mardinger O., Chaushu G., Nissan J. (2009). Characteristics of early versus late implant failure: A retrospective study. J. Oral Maxillofac. Surg..

[7] Guarnieri R., Zanza A., D’Angelo M., Di Nardo D., Del Giudice A., Mazzoni A., Reda R., Testarelli L. (2022). Correlation between peri-implant marginal bone loss progression and peri-implant sulcular fluid levels of metalloproteinase-8. J. Pers. Med..

[8] Guarnieri R., Miccoli G., Reda R., Mazzoni A., Di Nardo D., Testarelli L. (2022). Sulcus fluid volume, IL-6, and Il-1b concentrations in periodontal and peri-implant tissues comparing machined and laser-microtextured collar/abutment surfaces during 12 weeks of healing: A split-mouth RCT. Clin. Oral Implant. Res..

[9] Sinjari B., D’Addazio G., Traini T., Varvara G., Scarano A., Murmura G., Caputi S. (2019). A 10-year retrospective comparative human study on screw-retained versus cemented dental implant abutments. J. Biol. Regul. Homeost. Agents.

[10] Scarano A., Murmura G., Sinjiari B., Sollazzo V., Spinelli G., Carinci F. (2011). Analysis and structural examination of screw loosening in oral implants. Int. J. Immunopathol. Pharmacol..

[11] Manzano G., Montero J., Martín-Vallejo J., del Fabbro M., Bravo M., Testori T. (2016). Risk factors in early implant failure: A meta-analysis. Implant Dent..

[12] Palma-Carrió C., Maestre-Ferrín L., Peñarrocha-Oltra D., Peñarrocha-Diago M.A., Peñarrocha-Diago M. (2011). Risk factors associated with early failure of dental implants. A literature review. Med. Oral Patol. Oral Cir. Bucal.

[13] Cuschieri S. (2019). The STROBE guidelines. Saudi J. Anaesth..

[14] Casino A.J. (1998). Systemic factors contributing to implant failure. Oral Maxillofac. Surg. Clin. N. Am..

[15] Zinser M.J., Randelzhofer P., Kuiper L., Zöller J.E., de Lange G.L. (2013). The predictors of implant failure after maxillary sinus floor augmentation and reconstruction: A retrospective study of 1045 consecutive implants. Oral Surg. Oral Med. Oral Pathol. Oral Radiol..

[16] Conrad H.J., Jung J., Barczak M., Basu S., Seong W.J. (2011). Retrospective cohort study of the predictors of implant failure in the posterior maxilla. Int. J. Oral Maxillofac. Implant..

[17] Wallace S.S., Froum S.J. (2003). Effect of maxillary sinus augmentation on the survival of endosseous dental implants. A systematic review. Ann. Periodontol..

[18] Pabst A.M., Walter C., Ehbauer S., Zwiener I., Ziebart T., Al-Nawas B., Klein M.O. (2015). Analysis of implant-failure predictors in the posterior maxilla: A retrospective study of 1395 implants. J. Craniomaxillofac. Surg..

[19] Lozada J.L., Emanuelli S., James R.A., Boskovic M., Lindsted K. (1993). Root-form implants placed in subantral grafted sites. J. Calif. Dent. Assoc..

[20] Tran D.T., Gay I.C., Diaz-Rodriguez J., Parthasarathy K., Weltman R., Friedman L. (2016). Survival of dental implants placed in grafted and nongrafted bone: A retrospective study in a university setting. Int. J. Oral Maxillofac. Implant..

[21] Sesma N., Pannuti C., Cardaropoli G. (2012). Retrospective clinical study of 988 dual acid-etched implants placed in grafted and native bone for single-tooth replacement. Int. J. Oral Maxillofac. Implant..

[22] Olson J.W., Dent C.D., Morris H.F., Ochi S. (2000). Long-term assessment (5 to 71 months) of endosseous dental implants placed in the augmented maxillary sinus. Ann. Periodontol..

